# A Case of an Advanced Chain of Survival in Penetrating Cardiac Injury

**DOI:** 10.1155/2019/2895439

**Published:** 2019-07-02

**Authors:** Mads Jønsson Andersen, Frank V. De Paoli, Rikke Mærkedahl, Søren Vad Jepsen, Karoline Skov Dalgaard, Thomas Falstie, Gustav Gerstrøm

**Affiliations:** ^1^Department of Cardiology, Aarhus University Hospital, Denmark; ^2^Department of Cardiology, Herning Hospital, Denmark; ^3^Department of Thoracic Surgery, Aarhus University Hospital, Denmark; ^4^Department of Anesthesiology, Herning Hospital, Denmark; ^5^Prehospital, Medical Emergency Care Units, Central Denmark Region, Denmark; ^6^Department of Anesthesiology, Aarhus University Hospital, Denmark

## Abstract

The survival rate of penetrating cardiac trauma is dismal, with only a few patients reaching the hospital with any signs of life. Short transport time and close proximity to the trauma center are positive factors for survival. We report the successful case of a 21-year-old male with penetrating cardiac injury and tension-pneumothorax with long distance to a trauma facility. The patient was stabbed twice in the anterior left side of the thorax. The emergency services found the patient with suspicion of left tension-pneumothorax. Urgent left mini-thoracotomy was established resulting in spontaneous respiration and clinical improvement. Due to rapid clinical deterioration and clinical suspicion of pericardial tamponade, patient was transported to the local regional hospital only minutes away. Echocardiography confirmed tamponade, and urgent ultrasound-guided pericardiocentesis was performed. During the transport blood was intermittently drained from the pericardial sack until arrival at the trauma center where a penetrating injury to the left ventricle was repaired during urgent cardiac surgery. The patient was discharged 8 days after the incident.* Conclusion*. Well organized emergency medical transport systems increase the chance of survival in penetrating cardiac injuries. Urgent pericardiocentesis with continuous drainage can help stabilize a patient until arrival at trauma facility.

## 1. Introduction

Penetrating cardiac injury represents a true medical emergency. Despite modern treatment algorithms [1] mortality is still high. This case report illustrates the importance of rapid initial in scene management by the emergency response physician, urgent transport to closest emergency room for initial stabilization, pericardiocentesis, and transport to trauma center for definite treatment of multiple penetrating stab wounds to the thorax.

## 2. Case

We report a case of a 21-year-old Caucasian male with a penetrating cardiac injury due to stabbing. A rapid medical response team staffed by an anesthesiologist was dispatched and found the patient unresponsive and pale with two stab wounds above the left papilla, agonal gasps, no thoracic excursions, left hemithorax seeming elevated compared to the right side; barely palpable carotid pulse rate of approximately 100; and no major external bleeding. The initial assessment was a periarrest with suspicion of left tension-pneumothorax. Urgent left mini-thoracotomy was established via the 5th intercostal space resulting in spontaneous respiration with lung expanding while coughing and improved facial color with a palpable carotid pulse of above 120, and within minutes he regained consciousness. Reassessment raised suspicion of tamponade due to hypotension 85/60 mmHg and tachycardia; a FAST ultrasound from small portable ultrasound system confirmed the initial diagnosis with pericardial fluid, compressing right atrium and affecting the filling of the right ventricle. Closest regional hospital was a few minutes away; the nearest trauma center with thoracic surgical support was approximately 1 hour/100 km away. Due to the instability of the patient, he was transported to the nearby regional hospital without thoracic surgery competence capability. Patient was not intubated due to (a) risk of increasing thoracic pressure and further deteriorating of the patient and (b) short transport time. Upon arrival in the ER the clinical status of the patient had further deteriorated as he was unresponsive, tachycardic, and hypotensive with dilated neck veins. Clamshell thoracotomy was considered but instead an urgent ultrasound-guided pericardiocentesis was performed with insertion of pig-tail catheter. After initial removal of ≈ 100 mL of blood, the clinical situation improved and the patient was intubated. Clinical examination revealed no other injuries but continuous removal of blood from the pericardial space was needed in order to maintain mean arterial pressure above 50 mmHg. Due to lack of thoracic surgical expertise and since the hemorrhage could be controlled, the patient was transferred to the trauma center 100 km away. During transport, the patient was continuously resuscitated with plasma and packed red blood cells, while blood was intermittently removed from the pericardium in order to keep a mean arterial pressure around 50 mmHg. A total of 750 mL was removed in total during the hour long transport. Upon arrival at the trauma center patient was immediately prepped for surgery and a median sternotomy was performed. A 2 cm stab wound to the left ventricle was discovered (blood jetting), cardiorrhaphy (suturing the heart muscle) was performed, and hemostasis was secured. The heart and pericardial sack were inspected and no other pathologies were found. No further interventions were performed and the sternotomy was closed. Trauma CT revealed no other injuries. The patient's postoperative course was uneventful, and the patient was discharged 8 days after the incident.

## 3. Discussion

The present case demonstrates the importance of good collaboration between trauma teams in the prehospital setting, at the regional hospital and with the trauma center in order to resuscitate and transport a young and severely injured patient. Penetrating cardiac traumas carry a poor prognosis. In a 10-year consecutive cohort of chest trauma victims with penetrating cardiac injuries from a major Scandinavian trauma center, approximately 50% died before reaching the hospital. Of the patients reaching the trauma center, the mortality was 50% despite high intensity of care. A predictor for survival was signs of life in the emergency room [2]. Initial clinical examination together with analysis of the trauma mechanism will provide vital information on potential severity and location of the injury. In this case the clinical presentation of the patient, trauma mechanism, and objective signs led to urgent treatment [3]. If the extent of the injury remains undetermined and the condition of the patient permits, imaging can provide further information [4]. As in present case, tension-pneumothorax can be treated prehospitally by a mini-thoracotomy performed laterally in the fifth intercostal space. This incision can be extended to a clamshell thoracotomy for better access to both sides of the chest [5]. In this case clamshell thoracotomy performed on scene or in the emergency room at the regional hospital would probably have led to severe bleeding from the left ventricle and possible death. This procedure can be necessary in case of excessive bleeding, clotting of the pericardial catheter, and dislocation of the pericardial catheter. Cardiac tamponade after penetrating trauma may initially be managed by intermittent drainage and transfusions until a thoracotomy and cardiorrhaphy can be performed [6] even over long distances, as illustrated by this case.
